# Antimicrobial Screening and Fungicidal Properties of *Eucalýptus globulus* Ultrasonic Extracts

**DOI:** 10.3390/plants11111441

**Published:** 2022-05-28

**Authors:** Stanislav Sukhikh, Svetlana Ivanova, Olga Babich, Viktoria Larina, Olesia Krol, Alexander Prosekov, Alexander Popov, Olga Kriger

**Affiliations:** 1 Institute of Living Systems, Immanuel Kant Baltic Federal University, A. Nevskogo Street 14, 236016 Kaliningrad, Russia; stas-asp@mail.ru (S.S.); olich.43@mail.ru (O.B.); surinac@mail.ru (V.L.); ole-jolie@yandex.ru (O.K.); alexpo_kld@mail.ru (A.P.); olgakriger58@mail.ru (O.K.); 2 Natural Nutraceutical Biotesting Laboratory, Kemerovo State University, Krasnaya Street 6, 650043 Kemerovo, Russia; 3 Department of General Mathematics and Informatics, Kemerovo State University, Krasnaya Street 6, 650043 Kemerovo, Russia; 4 Laboratory of Biocatalysis, Kemerovo State University, Krasnaya Street 6, 650043 Kemerovo, Russia; a.prosekov@inbox.ru

**Keywords:** eucalyptus extracts, ultrasonic treatment, antimicrobial and fungicidal activity, biologically active substances, phytogenic feed additives

## Abstract

The prohibition of antibiotics has led to extensive research and use of phytogenic feed additives. James Barrie Kirkpatrick described four subspecies of eucalyptus (family Myrtaceae), including *Eucalýptus globulus*, in 1974. The maximum concentrations of quercetin-3D-glycoside (1703.30 g/mL), astragalin (1737.82 g/mL), chlorogenic acid (342.14 g/mL), catechin (282.54 g/mL), rosmarinic acid (36.39 g/mL), and 3,4-dihydroxybenzoic acid (27.55 g/mL) were found in samples of ultrasonic extraction with ethyl alcohol (extraction module 1:5, temperature of 32 °C, an ultrasonic exposure time of 25 min). Antimicrobial activity was observed in all studied samples after 12 h of incubation (against gram-positive (*Bacillus subtilis)* and gram-negative (*Pseudomonas aeruginosa)* bacteria, as well as representatives of yeast fungi (*Candida albicans*)); a more pronounced antimicrobial effect (lysis zone) was observed after ultrasonic processing of extracts for 20 and 25 min. *Bacillus subtilis, Pseudomonas aeruginosa*, and *Candida albicans* had lysis areas of 10.0 mm (20 min extraction with ultrasonic treatment), 13.0 mm (20 min extraction without ultrasonic treatment), and 15.5 mm (25 min extraction with ultrasonic treatment), respectively. *E. globulus* was demonstrated to be a source of biologically active phenolic compounds with antibacterial and fungicidal activity. More research on the use of *E. globulus* in feed additives is required.

## 1. Introduction

The use of a number of antibiotics in feed additives was only allowed until the end of 2005 after the European Union Regulation 1831/2003 on additives used in animal nutrition was published in 2003; in 2006, a large-scale study and application of phytogenic feed additives (PFAs) has commenced in Europe [1]. PFAs are considered the first alternative to antibiotic growth promoters (AGPs) based on their complex biological activity (antimicrobial, antioxidant and anti-inflammatory properties of biologically active plant compounds) [2,3]. The technology for the production of many medications and dosage forms, including tinctures, extracts, and juices, as well as hormones, enzymes, and antibiotics, is based on the process of isolating biologically active molecules from raw materials with a cellular structure. Released biologically active substances (BAS) are used as PFAs and herbal preparations in animal husbandry (to improve feed quality and safety), as well as additives that support animal health and well-being or act as immunomodulators, antioxidants, and digestive stimulants. These preparations can boost the productivity and quality of livestock products [3,4].

Essential oils, which are an integral part of PFAs, contain many biologically active substances of plants that accelerate the growth and weight gain of animals [5,6]. The antibacterial properties of PFAs are explained by the presence of phenolic compounds and their effect on pathogenic microorganisms [7]. The greatest effectiveness of essential oils is achieved by low pH, low temperature, and higher concentration [7].

There are numerous technologies for drying plant raw materials, such as microwave dehydration, which exposes the raw material to an intense electromagnetic field of microwave frequencies (MWF) [8]. The advantage of microwave drying over traditional drying is that heating occurs inside the dehydrated material throughout its volume and evenly in a relatively short time [9,10].

Infrared and acoustic (ultrasonic waves) drying of plant materials are also used [11]. Infrared drying can be carried out in large volumes and at high speed, at a temperature of 40–60 °C, preserving the content of biologically active substances up to 80–90% of the original plant material. Ultrasonic drying allows the raw material to be dried at any starting moisture content while preserving a high content of biologically active substances in the dried product [12,13]. A feature of this method is that the drying of raw materials proceeds without increasing the temperature, which makes this method suitable for drying heat-labile and easily oxidized materials [11].

In acoustic extraction, cavitation replaces molecular intracellular diffusion of plant material under the influence of ultrasound. Due to this phenomenon, the material is penetrated faster. The explosion of cavitation bubbles on the surface of the product results in a microjet that causes several effects such as surface flaking, erosion, and particle breakage. At the same time, the collapse of cavitation bubbles in a liquid medium leads to macroturbulence and micromixing [14,15,16].

Thorough grinding of plant materials leads to faster kinetics of extraction with ultrasound than even with microwave extraction [17,18]. The ultrasonic extraction method is very nuanced, causing the phenomenon of cavitation, it requires special conditions. The main advantages of ultrasonic extraction are lower energy costs, reduced solvent costs, and reduced extraction time [18]. Process parameters have a strong influence on extraction efficiency and should be carefully studied in the laboratory for any process in the pharmaceutical, cosmetic or food industry to obtain biologically active compounds from medicinal plant materials (MPMs) [19,20,21].

The key arguments for using herbal medications in treatment are their unique mechanisms of action, which have the potential to provide biocompatible and cost-effective treatments while also speeding up the discovery of novel drugs [22,23]. Plant-based natural products are the main source of new chemical compounds [24,25] that can be used in feed additives.

The emergence of multidrug resistance in antibiotic therapy is a frequent problem in veterinary medicine. Plant-derived active ingredients provide an alternative to synthetic antibiotics in the treatment of antibiotic-resistant infections. Many such phytochemicals have proven their antimicrobial and bactericidal potential [26,27,28].

In 1974, James Barrie Kirkpatrick described four subspecies of eucalyptus (family Myrtaceae), including *Eucalýptus globulus* [18]. There was a study that looked at the antibacterial and fungicidal properties of the *Eucalýptus globúlus* plant, which is an evergreen tree of the genus *Eucalýptus* of the Myrtaceae family. This plant is native to Australia. Aldoghaim et al. [28] и Pan et al. [20] demonstrated that secretory structures (essential oil receptacles) were discovered in the *Eucalýptus globúlus* leaves [20,29]. The *Eucalýptus globulus* leaves contain an essential oil (up to 2%), the main component of which is eucalyptol (1,8-cineol) [29]. Tannins, amarines, resins, terpene compounds, aldehydes, ketones, free and esterified alcohols, and carbonyl compounds are also found in essential oils [30]. Eucalyptol (1,8-cineole) is known for its mucolytic and antispasmodic, anti-inflammatory effects on the respiratory tract during disease (e.g., asthma) [31]. *Eucalýptus globúlus* oils have high antimicrobial (in particular, against gram-negative bacteria) and fungicidal activity [32,33].

This research aimed to study the antimicrobial and fungicidal properties of *E. globulus* extracts obtained with and without ultrasound.

## 2. Results

### 2.1. Determination of Dry Matter in E. globúlus Extracts

**Figure 1:**
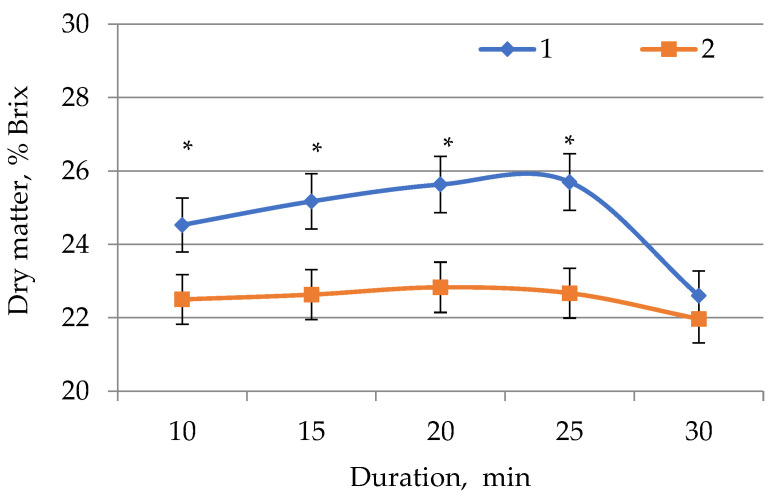
Dependence of the amount of dry matter in samples of *E. globúlus* extracts on the extraction time: 1—without US; 2—with US. Values followed by the symbol “*” do differ significantly (*p* < 0.05) as assessed by post hoc test (Tukey test).

Ultrasound (US) treatment of *E. globúlus* medicinal plant raw materials was performed with various hydromodules and durations. Each extract sample was tested for the amount of solids in percent. The results of studies on the effect of the duration of ultrasonic treatment of the medicinal plant *E. globúlus* on the yield of extractives at a concentration of 1:5 without and with the use of ultrasound are presented in Figure 1.

### 2.2. Study of the Antimicrobial Activity of E. globúlus Extracts

**Figure 2:**
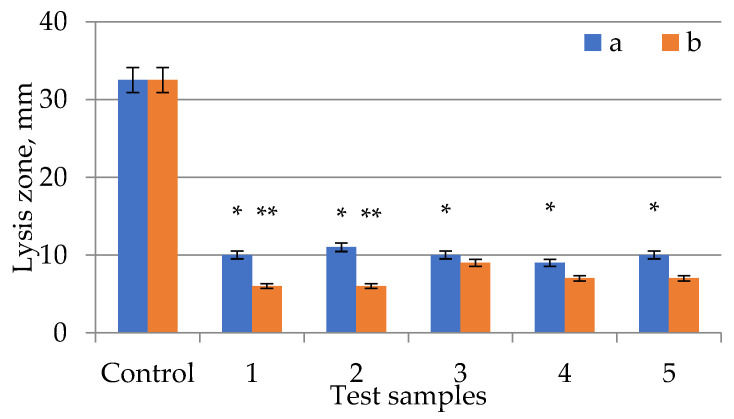
Testing the antimicrobial effect against *Bacillus subtilis* of *Eucalyptus globulus* extracts of different extraction times (a) with US treatment and (b) without US treatment: 1—10 min; 2—15 min; 3—20 min; 4—25 min; 5—30 min. Control—ampicillin (2.5 µg/10 µL). Values of the treated and control cultures followed by the symbol “*” do differ significantly (*p* < 0.05) as assessed by post hoc test (Tukey test). Values of the treated culture at same time followed by the symbol “**” do differ significantly (*p* < 0.05) as assessed by post hoc test (Tukey test). Values in columns/rows followed by the same letter do not differ significantly (*p* > 0.05) as assessed by post hoc test (Tukey test).

**Figure 3:**
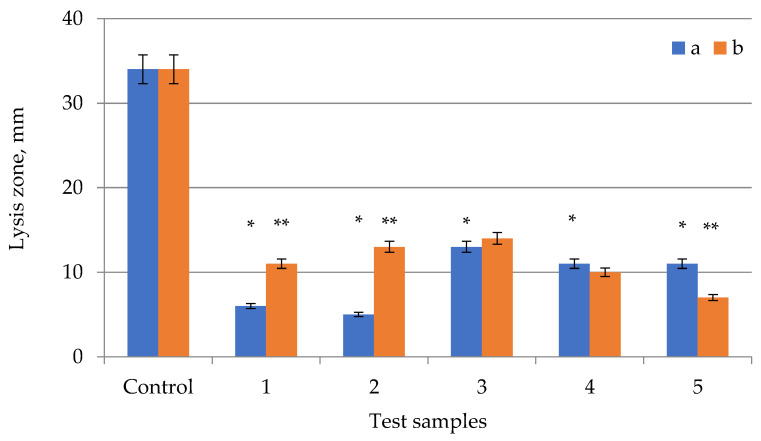
Testing the antimicrobial effect against *Pseudomonas* *aeruginosa* of *Eucalyptus globulus* extracts of different extraction times (a) with US treatment and (b) without US treatment: 1—10 min; 2—15 min; 3—20 min; 4—25 min; 5—30 min. Control—ampicillin (2.5 µg/10 µL). Values of the treated and control cultures followed by the symbol “*” do differ significantly (*p* < 0.05) as assessed by post hoc test (Tukey test). Values of the treated culture at same time followed by the symbol “**” do differ significantly (*p* < 0.05) as assessed by post hoc test (Tukey test).

**Figure 4:**
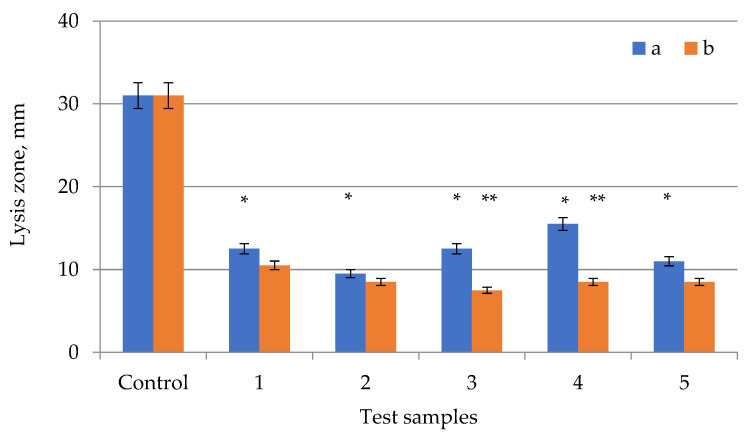
Testing the antimicrobial effect against *Candida albicans* of *Eucalyptus globulus* extracts of different extraction times (a) with US treatment and (b) without US treatment: 1—10 min; 2—15 min; 3—20 min; 4—25 min; 5—30 min. Control—fluconazole (2 mg/mL). Values of the treated and control cultures followed by the symbol “*” do differ significantly (*p* < 0.05) as assessed by post hoc test (Tukey test). Values of the treated culture at same time followed by the symbol “**” do differ significantly (*p* < 0.05) as assessed by post hoc test (Tukey test).

The obtained samples of *E. globúlus* extracts were tested for antimicrobial activity on several types of microorganisms (Figure 2, Figure 3 and Figure 4). Antimicrobial activity data are summarized in Table 1. Each test was carried out in duplicate, with the experiment being repeated if the result was positive.

*Candida albicans* is an opportunistic yeast that is widely distributed in the human intestinal flora. It can also survive outside the human body. It is found in the gastrointestinal tract and oral cavity in 40–60% of healthy adults. It is usually a commensal organism, but can become pathogenic in immunocompromised individuals under a variety of conditions.

A number of medications are used to treat fungal diseases, each with its own origin (natural or synthetic), spectrum and mechanism of action, antifungal effect (fungicidal or fungistatic), indications for use (local or systemic infections), and administration methods (orally, parenterally, externally) [34].

The antifungal effect of azoles, like polyene antibiotics, is due to a violation of the integrity of the fungal cell membrane, but the mechanism of action is different: azoles disrupt the synthesis of ergosterol, the main structural component of the fungal cell membrane. The effect is associated with inhibition of cytochrome P450-dependent enzymes, including 14-alpha-demethylase (catalyses the conversion of lanosterol to ergosterol), which leads to disruption of the synthesis of ergosterol in the fungal cell membrane.

Azoles (ketoconazole, fluconazole, itraconazole, voriconazole) have a wide spectrum of antifungal activity with a predominantly fungistatic effect. Azoles for systemic use are active against most pathogens of superficial and invasive mycoses, including *Cryptococcus neoformans*, *Coccidioides immitis*, *Histoplasma capsulatum*, *Blastomyces dermatitidis*, *Paraccoccidioides brasiliensis*. *Candida albicans*, *Candida glabrata*, *Candida krucei*, *Aspergillus* spp., *Fusarium* spp., and zygomycetes (class *Zygomycetes*) are usually resistant to azoles [33]. Thus, the antimicrobial activity of *E. globulus* metabolites was used.

### 2.3. Study of the Qualitative and Quantitative Composition of BASs in E. globulus Extracts

**Figure 5:**
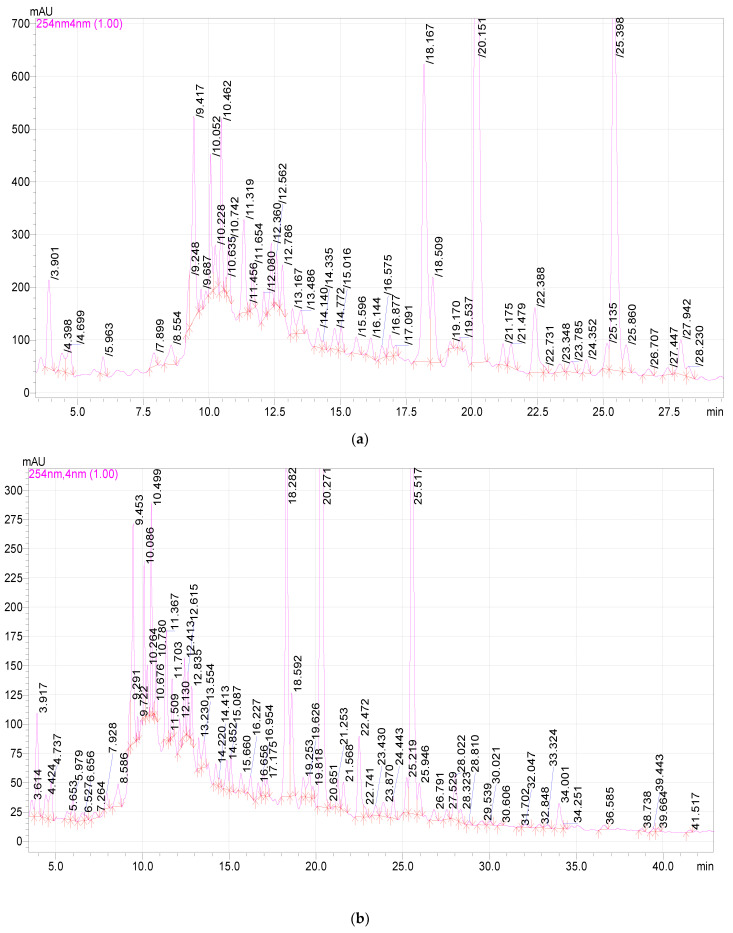

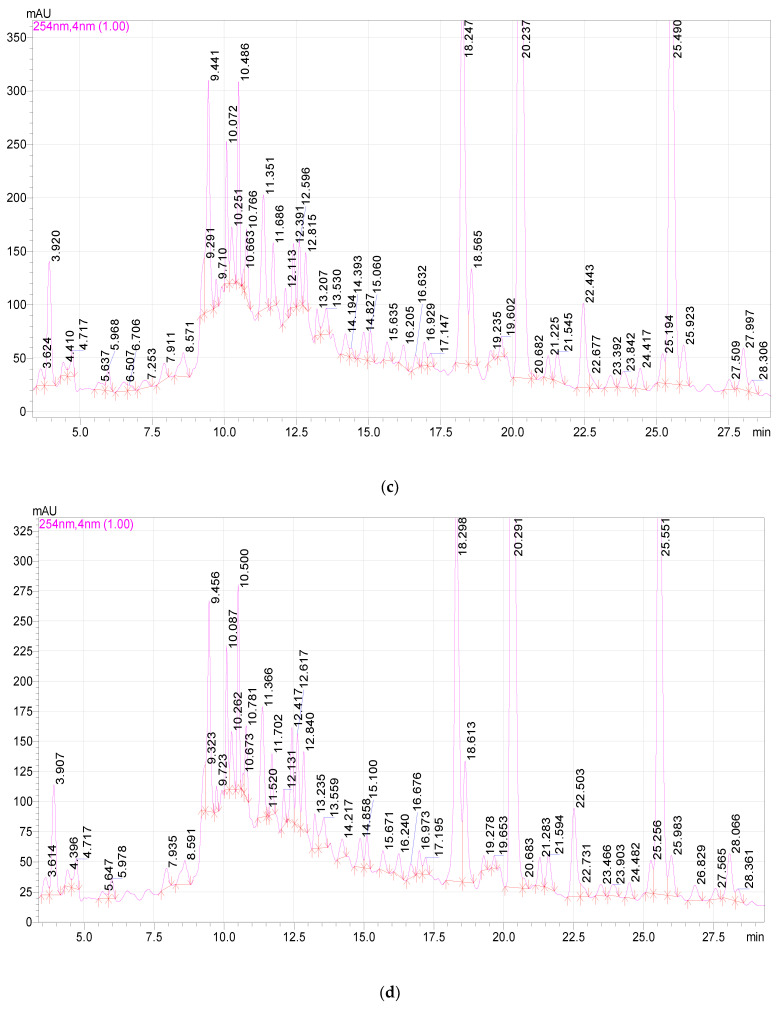

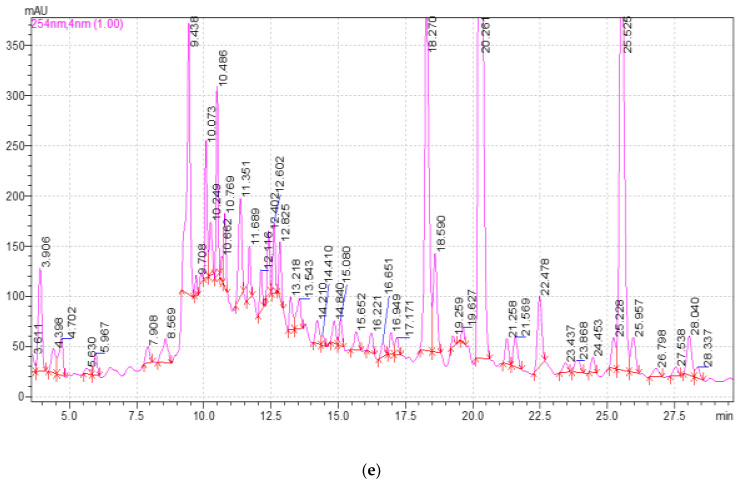
Chromatograms of extracts of different extraction times with ultrasonic treatment: (**a**) 10 min; (**b**) 15 min; (**c**) 20 min; (**d**) 25 min; (**e**) 30 min.

**Figure 6:**
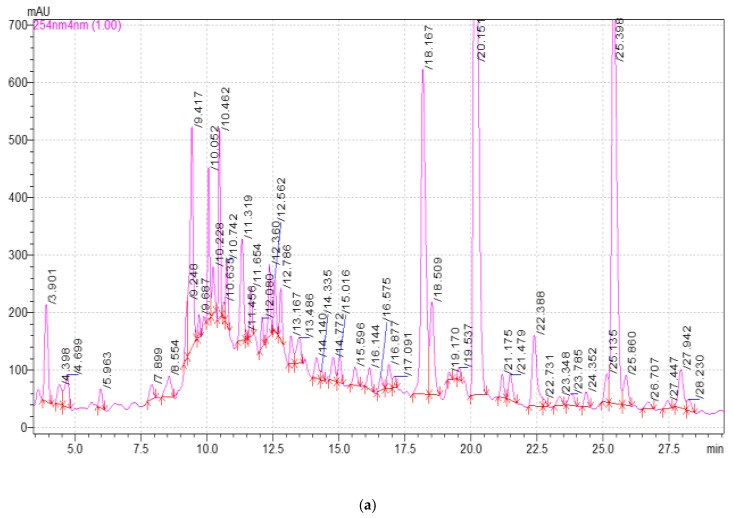

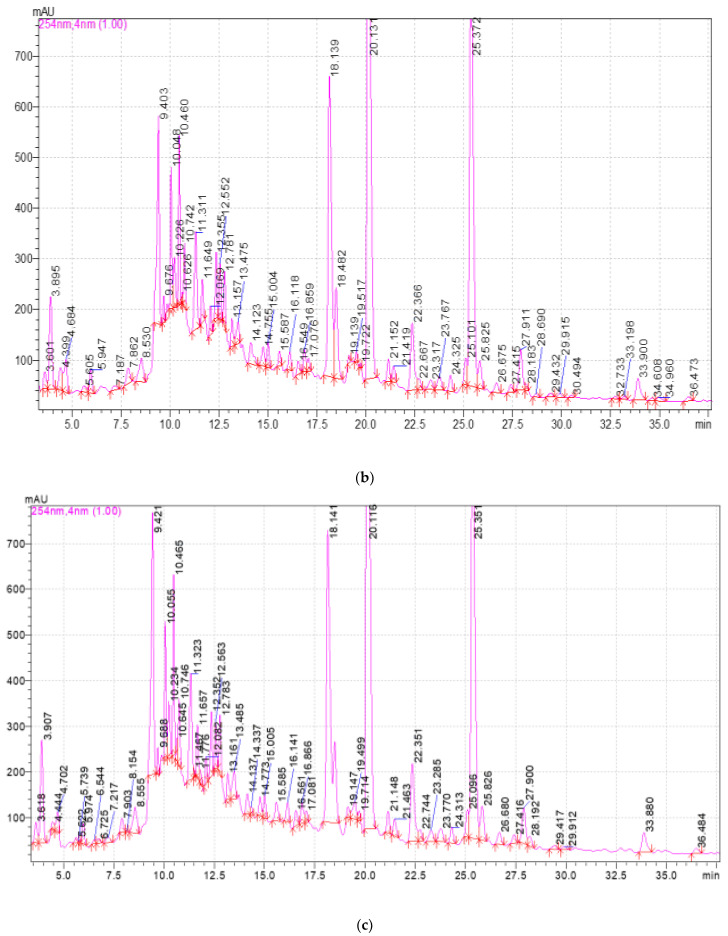

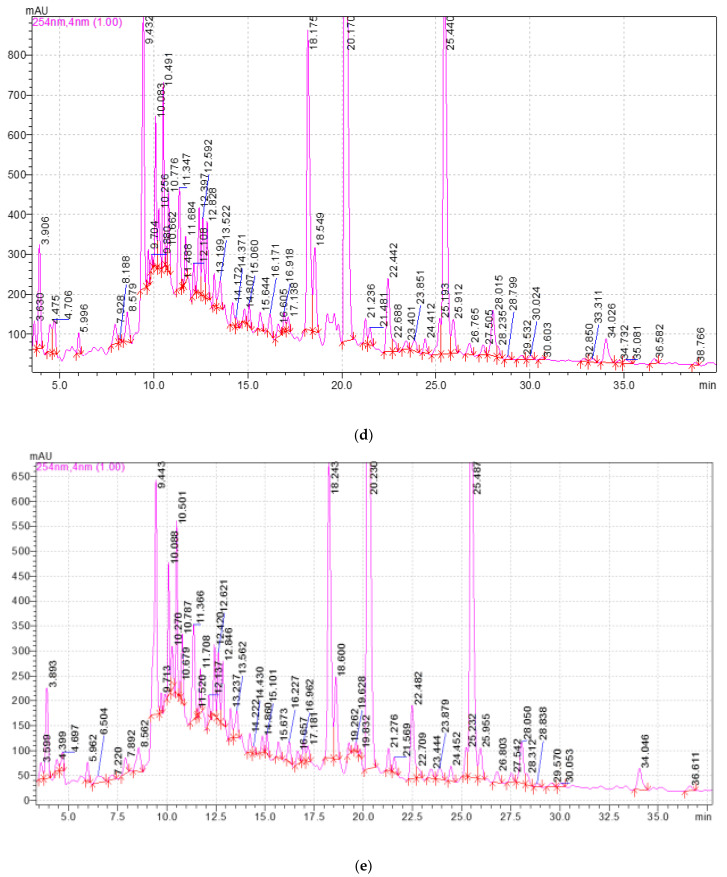
Chromatograms of extracts of different extraction times without ultrasonic treatment: (**a**) 10 min; (**b**) 15 min; (**c**) 20 min; (**d**) 25 min; (**e**) 30 min.

The HPLC results were obtained in the form of a chromatogram for each extract sample (Figure 5 and Figure 6).

The quantitative content of BASs found in extracts obtained with and without US treatment are presented in Table 2 and Table 3.

**Figure 7:**
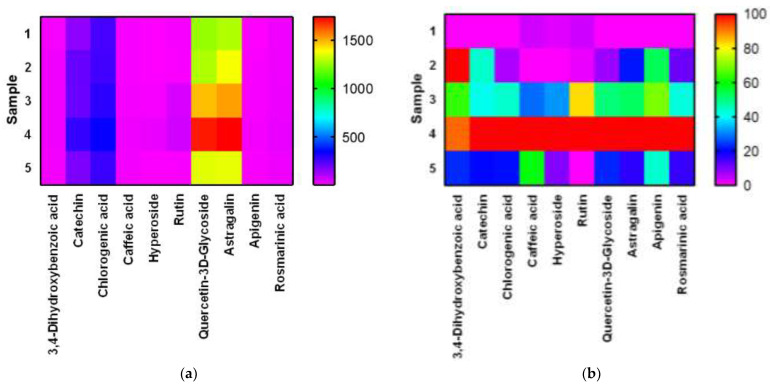
Heatmap for BAS content *E. globulus* extract with different extraction times (with US treatment): (**a**)—native data, (**b**)—after normalized.

For a visual presentation of the results of the table, we built a heat map in the Graph Pad Prism program. The Figure 7 shows heat maps for the original and normalized results. Normalization was carried out separately for each BAS.

**Figure 8:**
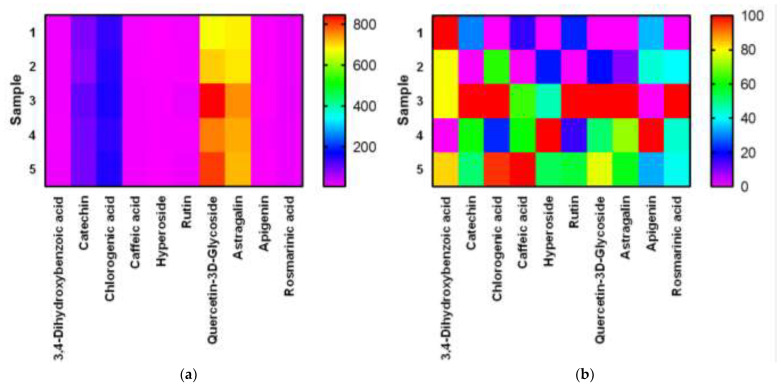
Heatmap for BAS content *E. globulus* extract with different extraction times (with US treatment): (**a**)—native data, (**b**)—after normalized.

For a visual presentation of the results of the table, we built a heat map in the Graph Pad Prism program. The Figure 8 shows heat maps for the original and normalized results. Normalization was carried out separately for each BAS.

**Figure 9:**
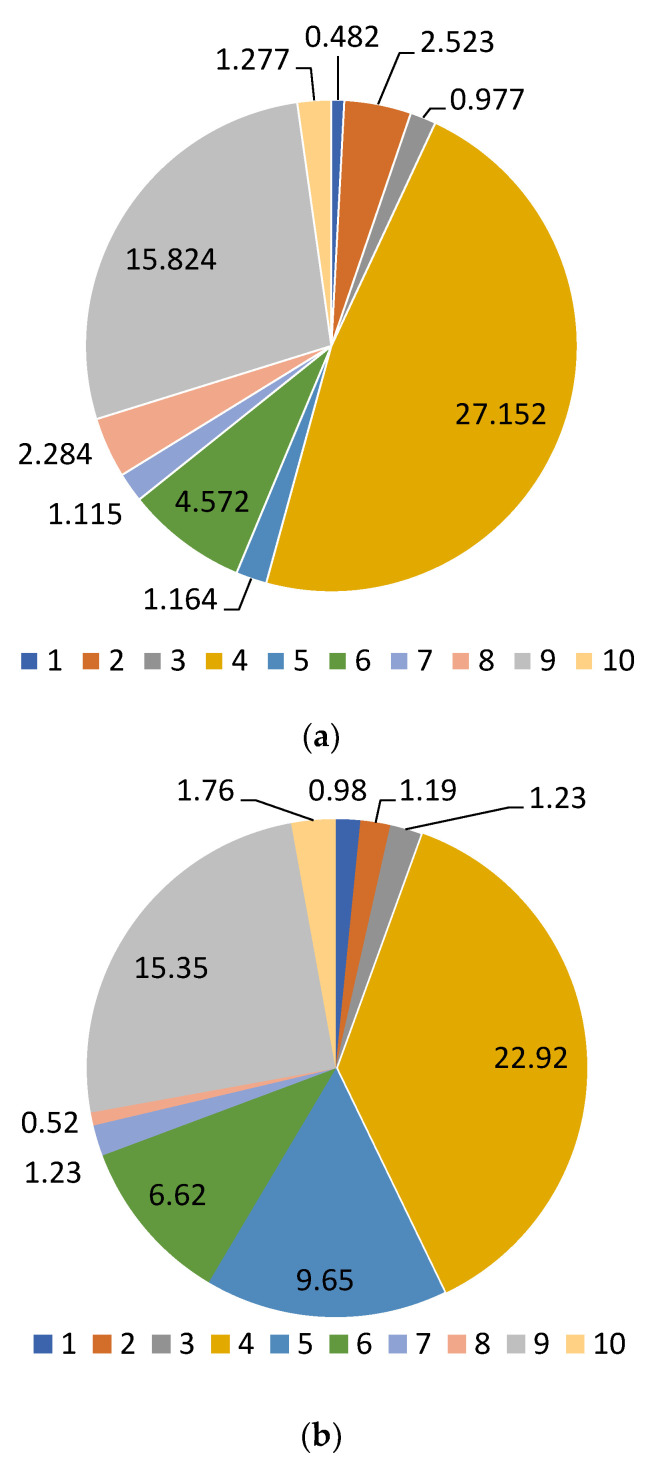
Percentage of identified BAS yield in extract samples (**a**) extraction for 25 min with US treatment; (**b**) extraction for 25 min without US treatment: 1—3,4-dihydroxybenzoic acid; 2—chlorogenic acid; 3—hyperoside; 4—quercetin-3D-glycoside; 5—apigenin; 6—cachetin; 7—caffeic acid; 8—rutin; 9—astragalin; 10—rosmarinic acid.

Figure 9 demonstrates the percentage of BASs found in extract samples (extraction for 25 min with and without ultrasonic treatment) in 5 g of dry plant materials and 25 mL of solvent.

## 3. Discussion

Figure 1 demonstrates that the samples with the highest dry matter content were obtained after ultrasonic treatment for 20 and 25 min, with quantitative yields of 25.63% and 25.70%, respectively. The least effective yield of extractive dry matter (24.53%) was observed after a 10-min ultrasonic treatment, which is insufficient [35]. However, a 30-min ultrasonic treatment yielded the lowest percentage of dry matter yield (22.60%). The yield of biologically active substances increased during ultrasonic extraction, peaking at 25 min and then declining (by 30 min). This fact is confirmed by the results of studies [36,37]. Similarly, during the extraction of samples without ultrasonic treatment for 10 and 15 min, an increase in the yield of dry extractive substances was observed (22.50% and 22.63%). The peak of the dry matter yield corresponded to the extraction for 20 min (22.83%). An increase in extraction time did not result in a significant decrease dry matter yield, especially if the extracted material was processed for 30 min. The results of these studies led to the conclusion that samples of eucalyptus extract with and without ultrasonic treatment showed similar dynamics of the accumulation of extractive solids depending on the duration of extraction, which is similar to the results of [38,39,40].

After 12 h of incubation of Petri dishes in a thermostat, all obtained samples of the extract demonstrated antimicrobial activity against microorganisms *B. subtilis* and *P. aeruginosa*. Over the same time period, no significant antimicrobial activity was observed against *E. coli*, possibly due to the bactericidal effect on this microorganism being very rapid. It was determined that the extract samples had antibacterial activity, and the extract sample after extraction with ultrasonic exposure for 20 min had the largest lysis zone (9 mm in diameter). In comparison, the control (antibiotic) had a lysis zone of 35 mm in diameter. *Candida albicans* are opportunistic yeasts and belong to the Sachoromycetes family. These fungi (yeasts) are endosymbionts, live on the surfaces of the skin and mucous membranes, and can sometimes cause a systemic infection. Human or animal infection occurs by changing the morphological form of *C. albicans* from blastospore to hyphae [31]. According to the results of studies [41], 70% methanol-water extract of eucalyptus showed significantly lower antimicrobial activity. The maximum lysis zone was 5.2 mm for *E. coli*. It was compared with the lysis zone of gentamicin, which was 7.3 mm for *E. coli*. The disk diffusion method was used to assess the antifungal activity of various concentrations of eucalyptus extracts in the study [42]. The ethanolic extract of eucalyptus caused a complete inhibition of the growth of the mycelium of *Fusarium* spp. at a concentration of 7–8 µL/mL after five days of incubation. The minimum inhibitory concentration and the minimum fungicidal concentration of the eucalyptus extract on the studied fungi were in the range of 7–8 µL/mL and 8–10 µL/mL, respectively. These results confirm the fungicidal properties of eucalyptus essential oils [42].

The extract samples (Figure 2, Figure 3 and Figure 4, Table 1) showed approximately the same lysis zones (from 9 mm to 11 mm in diameter), while ampicillin was almost three times more active. High antimicrobial activity was observed in extract samples with an extraction time of 15 min and ultrasonic treatment. All extract samples with ultrasonic treatment had an antimicrobial effect. The extract samples with the longest ultrasonic treatment time (20 min) had the strongest antibacterial effect, while extract samples with the shortest ultrasonic treatment time (30 min) had the weakest effect. The antibiotic had a lysis zone of 38 mm in diameter. It’s worth noting that the antibacterial efficiency of extract samples grew as the time of extraction with ultrasonic treatment increased (up to 20 min) and then declined after this peak. The antimicrobial activity of the dried extracts was empirically determined to be present on all disks. The largest lysis zone was found in extract samples treated with ultrasound for 20 min, the smallest zone—in samples treated for 10 and 15 min, and it did not change during ultrasonic treatment for 25 and 30 min.

The antifungal preparation showed high fungicidal activity, having an average lysis zone of 30.5 mm. Sample extracts also had antifungal activity. The largest lysis zone was observed in the extract samples after extraction with ultrasonic treatment for 10 min, according to the experiment. The lysis zone was 8.5 mm on average throughout the extracts. A weak fungicidal effect was revealed when testing a sample of the extract after extraction without ultrasonic treatment for 20 min—7.5 mm (Figure 4). Both the antifungal preparation and extract samples after extraction with ultrasonic treatment had fungicidal activity [41,42,43]. The lowest antifungal effect was observed in samples with extraction times ranging from 20 to 30 min without ultrasonic treatment. High fungicidal activity was found in extract samples after extraction with ultrasonic treatment for 25 min. The presence of antimicrobial activity was not confirmed when samples of the extract were tested on the gram-positive anaerobic bacillus *L. rhamnosus*.

Thus, the extract from this medicinal plant material had antimicrobial and fungicidal activities. The most pronounced effect was seen in extract samples with extraction times of 20 and 25 min and ultrasonic exposure. Eucalyptus extracts showed no antimicrobial activity against lactobacilli.

High performance liquid chromatography analysis (Figure 5 and Figure 6) found 3,4-dihydroxybenzoic acid, catechin, chlorogenic acid, caffeic acid, hyperoside, rutin, quercetin-3D-glycoside, astragalin, apigenin, and rosmarinic acid in *E. globulus* extract samples (Table 2 and Table 3). All detected substances have useful antioxidant properties [38,40]. The biologically active substances were found in all extract samples, but the highest concentration of these substances was observed in extract samples after 20 and 25 min of ultrasonic treatment. An average high content of quercetin-3D-glycoside (from 1283.82 to 1703.30 µg/mL), astragalin (from 1283.64 µg/mL to 1737.82 µg/mL), chlorogenic acid, and catechin was observed in extract samples after extraction with ultrasonic treatment. Caffeic acid, 3,4-dihydroxybenzoic acid, hyperoside, rutin, apigenin, and rosmarinic acid were minimally detected (Table 2). Extract samples subjected to extraction with ultrasound for 15 min showed the highest content of 3,4-dihydroxybenzoic acid among samples with different ultrasonic treatment times. The high content of the antioxidant astragalin was found in the extract samples with the duration of ultrasound extraction of 20 and 25 min, the lowest with the duration of plant material treatment for 10 min. This flavonoid has an anti-inflammatory effect, as well as antioxidant activity [34]. The maximum concentration of rutin was found in extract samples with an ultrasonic (US) extraction time of 20 min. Rutin is a glycoside that combines the properties of the flavonol quercetin and the disaccharide rutinoside, which belongs to the P vitamins. Rutin exhibits pharmacological properties (anti-inflammatory, antiallergic) [44]. Rosmarinic acid in extract samples (extraction time with ultrasonic treatment for 25 min) was isolated in an amount of 36.39 µg/mL, and this is its maximum yield in this experiment. Rosmarinic acid is an ester consisting of caffeic acid and 3,4-dihydroxyphenyl lactic acid. Rosmarinic acid exhibits antioxidant, antiviral, antibacterial, and antitumor activities [45]. Some studies have shown that rosmarinic acid has a light protective factor against UV radiation [46]. Analyzing the obtained data for samples after extraction without US treatment, it can be concluded that extraction for 20 and 25 min will be the most effective. The highest yield of biologically active substances from *E. globulus* leaves was observed when using these treatment regimes.

Our data on the qualitative and quantitative composition of *E. globulus* BASs are consistent with the results of the study [41]. The study [41] showed that *E. globulus* extracts contain 3,4-dihydroxybenzoic acid in the amount of 14.39 µg/mL, catechin in the amount of 90.48 µg/mL, chlorogenic acid in the amount of 200.38 µg/mL, caffeic acid in the amount of 20.41 µg/mL, hyperoside in the amount of 21.73 µg/L, rutin in the amount of 48.65 µg/mL, quercetin-3 D-glycoside in the amount of 38.95 µg/mL, apigenin in the amount of 2.85 µg/mL, rosmarinic acid in the amount of 9.85 µg/mL. According to experimental data, we can conclude that the amounts of biologically active substances obtained by us during extraction with ultrasonic treatment for 25 min exceed the content of the components obtained by the authors of [41], except for rutin and apigenin. The content of apigenin exceeds the values obtained by us by 6.1%, and that of rutin by 31.4%. Astragalin, presented in [41], was not found in our study of the composition of the eucalyptus extract. A significant difference in the quantitative and qualitative composition of biologically active substances can be explained by the fact that scientists studied 70% methanol-water extract without ultrasonic treatment.

The concentrations of extractives contained in a sample of the dry mass of *E. globulus* are presented in Table 2 and Table 3. A gradual increase in the yield of BASs was observed with an increase in the duration of extraction with ultrasonic treatment from 10 min, reaching a peak yield at 25 min of extraction. With an increase in the duration of extraction to 30 min, the yield of biologically active substances decreased, especially in the case of astragalin and quercetin-3D-glycoside. There was a downward difference of the concentration of biologically active substances in the studied samples of the extract without ultrasonic treatment. The highest yield of BASs in extract samples obtained without US treatment was observed after 25-min extraction. This is because cavitation destroys the molecules of useful substances during long-term processing, resulting in an extract containing a high concentration of ballast substances. The duration of ultrasonic treatment appears to have a different effect on both the release rate and the preservation of the activity of the isolated components. The recommended time of ultrasonic extraction for the release of the maximum amount of BASs is 25 min, for extraction without ultrasonic treatment—20 min.

Both options can be used to produce phytogenic feed additives; however, sample extraction using ultrasound is more efficient (reduced diluent costs, more useful BASs). Extraction by ultrasonic treatment for 25 min is recommended for the production of a liquid extract of *E. globulus* in order to yield the maximum amount of BASs. With these parameters, the yield of extractive substances will be quite high, even in comparison with 70% methanol-water extracts of eucalyptus studied in the study [41].

## 4. Materials and Methods

### 4.1. Object of Research

Dry *Eucalýptus globúlus* leaves (FARMGROUP, Barnaul, Russia) were purchased in a pharmacy chain. Eucalyptus leaves were harvested in accordance with GOST R 59425-2021 (Organic products made from wild raw material, introduced on 1 June 2021). Batch number M7-16/2020, shelf life 3 years, storage conditions: temperature 15–25 °C, protection from moisture and light, out of reach of children. During the study, this type of medicinal plant material was subjected to ultrasonic treatment of set intensity and varying durations, after which the isolated product was evaluated for dry matter content according to the 12th edition of the Russian Federation’s State Pharmacology, antimicrobial resistance, and the presence of biologically active substances using the chromatographic method.

### 4.2. Research Methods

Ethyl alcohol was chosen as the solvent (extractant).

Eucalyptus extract was prepared at a concentration of 1:5. The required amount of extractant was calculated using the formula:

$$V_{\mathrm{ext}}=V_{\mathrm{tin}}+m_{\mathrm{rm}}\times C_{a},$$

where V_ext_—amount of extractant, mL; V_tin_—given amount of tincture, mL; m_rm_—initial amount of raw materials, g; C_a_—absorption coefficient.

To obtain 15 mL of 0.2% eucalyptus tincture, 25 mL of 70% ethanol and 5 g of dried *E. globúlus* leaves were used. The leaves of plant raw materials were cut with scissors to 3–5 mm. These optimal dimensions preserve the cellular structure of the starting material; diffuse processes will prevail, slowing the extraction; nevertheless, the resulting extract will be easier to purify. Then the resulting cut medicinal plant material was weighed in five repetitions on a Pioneer laboratory electronic balance (DV-expert, Moscow, Russia). The resulting mass of raw material was filled with a prepared in advance 70% ethanol (25 mL) to obtain a concentration of 1:5. The extraction material was well mixed to ensure that all dry raw materials were soaked, and it was infused at room temperature for 2 h.

Two flasks containing extraction material were secured on a holder and placed in an ultrasonic bath with heating Bandelin RK 102 H, Sonorex Super (Nikolab, Moscow, Russia); they were lowered so that water completely covered the level of extraction material inside the flask. During ultrasonic treatment, the medium was heated up to 32 °C [29]. The ultrasound action oscillation frequency was 20–22 kHz, and the intensity was 1–70 W/cm^2^ [26]. The material was extracted at various time intervals of 10, 15, 20, 25, and 30 min of processing.

After extraction, the material was filtered through a filter and infused in a two-chamber Bosch KAH92LQ25R refrigerator (Robert Bosch GmbH, Stuttgart, Germany) for 48 h at a temperature of 6–8 °C until a clear liquid was obtained. The solubility and precipitation of ballast substances decreased at this temperature. Following settling, filtration was repeated. The resulting preparation was labeled and stored in a vial in a refrigerator at a temperature of 10–15 °C.

Further studies were carried out on the quality indicators of the obtained samples of extracts. The samples were tested for the presence of dry substances, biologically active substances, antimicrobial and fungicidal activity.

### 4.3. Evaluation of the Quality of Eucalýptus globúlus Leaf Extract

#### 4.3.1. Determination of Dry Residue by Refractometric Method

The obtained filtered samples of the extracts were tested for dry matter content. Before testing on a HI96800 digital refractometer (Eltemix, Krasnodar, Russia), a test was performed using distilled water—0.0% Brix. The ambient temperature did not go beyond (20 ± 2) °C.

After zeroing, 2–3 drops of the test solution were applied to the sample cell, and the dry matter content in % Brix was obtained in 1.5 s. After the measurement, the residues were removed with filter paper, and the cell was washed with distilled water. Each of the extract samples was tested in this way.

#### 4.3.2. Method for Determining the Antimicrobial Activity of a Plant Extract

The disk-diffusion method was used to determine the antimicrobial effect of all obtained samples of extracts from medicinal plant materials. All samples of obtained *E. Globulus* extracts of were tested. All microorganisms were purchased from Scientific Center “Kurchatov Institute”—Research Institute for Genetics and Selection of Industrial Microorganisms (Moscow, Russia). Three types of pure cultures of microorganisms were used for testing: gram negative facultatively anaerobic rods *Escherichia* *coli* ATCC25922, aerobic *Pseudomonas aeruginosa* ATCC27853, gram positive facultatively anaerobic *Bacillus subtilis* ATCC6633, and *Lactobacillus rhamnosus* ATCC53103.

Ampicillin (Sintez, Moscow, Russia) was chosen as a positive control for bacteria, since this drug of the penicillin group has a pronounced natural antibacterial effect, which is associated with impaired synthesis of cell wall components by blocking the transpeptidation reaction during the synthesis of peptidoglycan (murein). This mechanism of antibacterial action is apparently inherent in *E. globulus* extracts, according to investigations [29,30]. The ampicillin concentration was 2.5 µg/10 µL. An aqueous solution of the antibiotic ampicillin (white powder) was used to prepare a solution for intramuscular administration in order to compare the antimicrobial activity of plant extracts [33].

The antimicrobial activity of eucalyptus extracts against *Candida albicans* was studied on Sabouraud agar (Microlab, Moscow, Russia) for 12 h. To compare the lysis of fungicidal activity, the antifungal preparation Fluconazole-Vertex (Vertex, St. Petersburg, Russia) was used. Fuconazole concentration was 2 mg/mL. Fluconazole-Vertex synthesizes the antifungal enzyme 14-α-demethylase. The preparation is effective against mycoses, in particular, mycoses caused by *Candida albicans*. The culture of microorganisms was carried out under sterile conditions in a class 2 microbiological safety cabinet “Laminar-S”-1.2 Neoteric (Amedix-engineering, Nizhny Novgorod, Russia).

Specific nutrient media were prepared and used for each species of microorganism: LB (lysogeny broth) medium for *E. coli*, *B. subtilis*, and *P. aeruginosa*; MRS medium for *L. rhamnosus*. These microorganisms were preliminarily cultivated in a clean medium at a temperature of 37 °C for 24 h [30].

Antimicrobial activity of eucalyptus extracts was tested, including against *Candida albicans* ATCC 10231.

A pure culture of *C. albicans* was used in the experiment. *Candida albicans*, removed with a spatula under sterile conditions, was introduced into Sabouraud liquid sterile nutrient medium (Mikrolab, Moscow, Russia) based on dextrose and peptone, and tightly sealed. The culture was incubated in a Binder BD 240 thermostat (DV-expert, Moscow, Russia) for 48 h at a temperature of 37 °C. After cultivation, the culture was stored in a refrigerator at 3–8 °C.

An aqueous solution of the antibiotic ampicillin (white powder) was used to prepare a solution for intramuscular administration in order to compare the antimicrobial activity of plant extracts [33]. The antimicrobial activity of eucalyptus extracts against *C. albicans* was studied on Sabouraud agar (Microlab, Moscow, Russia) for 12 h. To compare the lysis of antimicrobial activity, the antifungal preparation Fluconazole-Vertex (Vertex, St. Petersburg, Russia) was used. Fluconazole-Vertex synthesizes the antifungal enzyme 14-α-demethylase. The preparation is effective against mycoses, in particular, mycoses caused by *C. albicans*.

The culture of microorganisms was carried out under sterile conditions in a class 2 microbiological safety cabinet “Laminar-S”-1.2 Neoteric (Amedix-engineering, Nizhny Novgorod, Russia).

The prepared suspension of bacteria was tested for optical density on a KFK-3 photoelectric colorimeter (ZOMZ, Sergiev Posad, Russia), after which it was applied to solid media in petri dishes in an amount of 175 µL (for *E. coli* and *P. aeruginosa*) and 200 µL (for *B. subtilis*), then spread evenly with a spatula in the continuous lawn technique over the entire surface of the agar until absorbed into the solid agar. The culture of C. albicans had a high optical density, at a wavelength of 600 nm 1.6 × 10^8^ CFU/mL. The suspension of *C. albicans* was applied to Sabouraud agar in an amount of 100 µL.

Pre-prepared disks impregnated with 10 µL per disk with an extractant (70% ethyl alcohol) and extracts of 10 µL each, as well as disks impregnated with an antibiotic solution, after inoculation of microorganisms, were placed in the marked zones one at a time: test disks with an extractant, 2–4 discs in each well, a disc with the *E. globulus* extracts and a disc impregnated with an antibiotic. A disc impregnated with an antibiotic and an antifungal drug was placed last for innoculation. Next, the plates were placed in a thermostat with an internal temperature of 37 °C for 12 h exactly. At the end of the incubation period, the dishes were removed from the thermostat (Laboratornoye osnashcheniye, Moscow, Russia), and the diameter of the lysis zone around the disk was measured.

#### 4.3.3. Methodology and Conditions for High-Performance Liquid Chromatography

HPLC analysis was carried out on a LC-20AB “Shimadzu” Prominence chromatograph with a binary pump (Shimadzu, Kyoto, Japan), Zorbax 300SB-C18 4.6*250vv 5 µm column (Agelent, Melbourne, Australia), SPD-M20A diode array detector (Agelent, Melbourne, Australia).

The separation was carried out at a temperature of 35 °C in the gradient elution mode. Mobile phase: eluent A—0.1% trifluoroacetic acid in bidistilled water, B—acetonitrile. The sample volume was 5 µL. The flow rate was 1 mL/min, the analytical wavelength was 254, 280, and 325 nm. The retention times and spectra of individual standard substances were used to identify the extract components. The concentration of compounds was calculated from the calibration equations. The error in determining the concentration was 5%.

Identification was carried out according to absorption spectra and exit times of individual standards: cafftaric acid (CAS: 67879-58-7), chlorogenic acid (CAS: 327-97-9), caffeic acid (CAS: 331-39-5), coumaric acid (CAS: 501-98-4), ferulic acid (CAS: 1135-24-6), chicoric acid (CAS: 70831-56-0), rosmarinic Acid (CAS: 20283-92-5), hyperoside (CAS: 482-36-0), astragalin (CAS: 480-10-4), apigenin (CAS: 520-36-5), rutin (CAS: 153-18-4), catechin (CAS: 7295-85-4), hesperitin (CAS: 520-26-3), quercetin-3D-glucoside (CAS: 482-35-9), 3,4-dihydroxybenzoic acid (CAS: 99-50-3).

All chemicals (analytical or higher grade) used in this study were purchased from Fluka/Sigma-Aldrich (Sigma-Aldrich Rus, Moscow, Russia).

### 4.4. Statistical Analysis

Each experiment was repeated three times, and the data are expressed as means ± standard deviation. Data processing was carried out via the standard methods of mathematical statistics. Post hoc analysis (Tukey test) was undertaken to identify samples that were significantly different from each other. The equality of the variances of the extracted samples was checked using the Levene test. The data were subjected to analysis of variance (ANOVA) using Statistica 10.0 (StatSoft Inc., 2007, Tusla, OK, USA). Differences between means were considered significant when the confidence interval was below 5% (*p* < 0.05).

## 5. Conclusions

The extraction of BASs from medicinal plant materials is essential in the production of plant-based medicines. The prohibition of the uncontrolled use of antibiotic preparations was the primary impetus for the development of biotechnologies in the production of phytogenic feed additives and herbal preparations.

The influence of several parameters on the yield of dry matter during ultrasonic extraction was studied. The total yield of *E. globulus* dry matter was found to be highest (25.70%) after 25-min extraction with ultrasonic treatment and lowest after 30-min extraction (22.2%). Extraction for 10 and 15 min is insufficient, as an increase in dry matter yield was observed. US treatment had a positive effect on the dry matter yield, in comparison with extracts obtained without US treatment. After 12 h of incubation, antimicrobial activity was observed in all studied extract samples. After extraction with ultrasonic treatment for 20 and 25 min, the extracts had a more pronounced antimicrobial effect (lysis zone). The quantitative yield of BASs from eucalyptus extract samples after extraction with ultrasonic treatment was almost two times higher than in extract samples obtained without it. The recommended time of US exposure is 25 min.

Eucalyptus extracts are widely used for the production of phytogenic feed additives [46]. The natural eucalyptus oils included in the feed additive improve the functioning of the respiratory organs of animals and birds, facilitate breathing, have an antispasmodic, expectorant, anti-inflammatory, vasoconstrictive, analgesic effect, stimulate water and feed intake, improve metabolism and reduce the negative effect of heat stress, have adaptogenic effect under unfavorable environmental conditions and under conditions of extreme stress, reduce the number of post-vaccination complications, and have a moderate antibacterial effect [46]. The period of extraction with ultrasonic treatment of medicinal plant materials should not exceed 25 min to produce an effective phytogenic feed additive based on *E. globúlus* (the yield of useful active substances was the highest with strong antiseptic properties). Although the antimicrobial properties of *E. globúlus* dry extract do not claim to be equivalent to those of the strongest synthetic antibiotics, phytogenic feed additives based on eucalyptus can be used as a substitute for antimicrobial feed additives. However, more research into the nutritional value and digestion of feed by animals in industrial settings is required.

## Figures and Tables

**Table 1:** Inhibition zone of *Eucalyptus globulus* extracts and ampicillin against various strains of microorganisms.

Extraction Method		Extraction Time, min	Microorganism Strain		
			*Bacillus subtilis*	*Pseudomonas* *aeruginosa*	*Candida albicans*
	Without ultrasonic treatment	10	6.0	11.0	10.5
		15	6.0	13.0	8.5
		20	9.0	14.0	7.5
		25	7.0	10.0	8.5
		30	7.0	7.0	8.5
	With ultrasonic treatment	10	10.0	6.0	12.5
		15	11.0	5.0	9.5
		20	10.0	13.0	12.5
		25	9.0	11.0	15.5
		30	10.0	11.0	11.0
	Ampicillin	-	32.5	34.0	31.0
	Fluconazole	-	-	-	31.0

**Table 2:** Concentration of substances (µg/mL) in samples of *E. globulus* extract with different extraction times (with US treatment).

Component	Release Time, min	Extract Samples				
		1	2	3	4	5
3,4-dihydroxybenzoic acid	5.980	19.16 ± 0.2 ^a^	28.30 ± 0.2 ^b^	25.00 ± 0.2 ^b^	27.55 ± 0.2 ^b^	21.28 ± 0.2 ^a^
Catechin	9.689	157.86 ± 1.2 ^a^	212.64 ± 1.2 ^b^	209.52 ± 1.2 ^b^	282.54 ± 1.2 ^c^	183.19 ± 1.2 ^d^
Chlorogenic acid	10.464	256.46 ± 1.2 ^a^	261.88 ± 1.2 ^a^	294.16 ± 1.2 ^ab^	342.14 ± 1.2 ^b^	274.38 ± 1.2 ^a^
Caffeic acid	10.727	17.96 ± 0.2 ^a^	17.64 ± 0.2 ^a^	20.17 ± 0.2 ^a^	26.54 ± 0.2 ^b^	22.90 ± 0.2 ^ab^
Hyperoside	19.537	9.51 ± 0.1 ^a^	8.90 ± 0.1 ^a^	16.67 ± 1.2 ^b^	33.38 ± 0.2 ^c^	11.26 ± 0.2 ^a^
Rutin	19.537	16.16 ± 0.2 ^a^	15.03 ± 0.2 ^a^	60.13 ± 0.2 ^b^	69.79 ± 0.1 ^b^	14.07 ± 0.2 ^a^
Quercetin-3D-Glycoside	20.151	1246.82 ± 12.3 ^a^	1281.48 ± 12.3 ^a^	1477.78 ± 12.3 ^b^	1703.30 ± 12.3 ^c^	1350.34 ± 12.3 ^ab^
Astragalin	25.398	1283.64 ± 12.3 ^a^	1381.04 ± 12.3 ^a^	1521.44 ± 12.3 ^b^	1737.82 ± 12.3 ^c^	1358.68 ± 12.3 ^a^
Apigenin	26.710	7.42 ± 0.1 ^a^	14.26 ± 0.2 ^b^	16.36 ± 0.2 ^bc^	20.28 ± 0.2 ^c^	13.04 ± 0.2 ^b^
Rosmarinic acid	28.191	26.22 ± 1.2 ^a^	27.4 ± 0.2 ^a^	30.56 ± 0.2 ^ab^	36.39 ± 0.2 ^b^	27.83 ± 0.2 ^a^

1—10 min extraction; 2—15 min extraction; 3—20 min extraction; 4—25 min extraction; 5—30 min extraction. Values in rows followed by the same letter do not differ significantly (*p* > 0.05) as assessed by post hoc test (Tukey test).

**Table 3:** Concentration of substances (µg/mL) in samples of *E. globulus* extract with different extraction times (without US treatment).

Component	Release Time, min	Extract Samples				
		1	2	3	4	5
3,4-dihydroxybenzoic acid	5.980	13.98 ± 0.2 ^a^	13.25 ± 0.2 ^a^	13.26 ± 0.2 ^a^	10.44 ± 0.2 ^a^	13.39 ± 0.2 ^a^
Catechin	9.689	87.34 ± 0.2 ^a^	79.77 ± 0.2 ^b^	105.09 ± 1.2 ^c^	95.15 ± 0.2 ^a^	92.75 ± 0.2 ^a^
Chlorogenic acid	10.464	136.12 ± 1.2 ^a^	145.93 ± 1.2 ^ab^	151.70 ± 1.2 ^b^	139.67 ± 1.2 ^a^	151.09 ± 1.2 ^b^
Caffeic acid	10.727	9.00 ± 0.1 ^a^	8.56 ± 0.1 ^a^	10.34 ± 0.2 ^a^	10.22 ± 0.2 ^a^	11.32 ± 0.2 ^a^
Hyperoside	19.537	4.80 ± 0.1 ^a^	5.26 ± 0.1 ^a^	5.78 ± 0.1 ^a^	6.94 ± 0.1 ^a^	5.94 ± 0.1 ^a^
Rutin	19.537	9.19 ± 0.1 ^a^	7.22 ± 0.1 ^a^	16.09 ± 0.2 ^b^	8.60 ± 0.1 ^a^	12.16 ± 0.2 ^b^
Quercetin-3D-Glycoside	20.151	672.92 ± 1.2 ^a^	708.34 ± 1.2 ^a^	845.28 ± 1.2 ^b^	761.78 ± 1.2 ^b^	806.94 ± 1.2 ^b^
Astragalin	25.398	686.00 ± 1.2 ^a^	692.06 ± 1.2 ^a^	750.88 ± 1.2 ^b^	732.50 ± 1.2 ^ab^	723.80 ± 1.2 ^ab^
Apigenin	26.710	5.09 ± 0.1 ^a^	5.48 ± 0.1 ^a^	3.56 ± 0.1 ^b^	8.01 ± 0.1 ^c^	5.05 ± 0.1 ^a^
Rosmarinic acid	28.191	14.44 ± 0.2 ^a^	15.18 ± 0.2 ^a^	16.29 ± 0.2 ^a^	15.25 ± 0.2 ^a^	15.20 ± 0.2 ^a^

1—10 min extraction; 2—15 min extraction; 3—20 min extraction; 4—25 min extraction; 5—30 min extraction. Values in rows followed by the same letter do not differ significantly (*p* > 0.05) as assessed by post hoc test (Tukey test).

## Data Availability

The data are included in the manuscript.
